# Integrative Bioinformatics Approaches in Environmental Biotechnology: A Review

**DOI:** 10.1155/tswj/3495506

**Published:** 2026-08-06

**Authors:** Yohannes Tsegay Teklay

**Affiliations:** ^1^ Faculty of Biotechnology, Mekelle Institute of Technology, Mekelle University, Mekelle, Ethiopia, mu.edu.et

**Keywords:** bioremediation, biotechnology, environmental bioinformatics, genomics, multiomics integration, predictive modeling

## Abstract

Environmental biotechnology increasingly relies on bioinformatics to address global challenges in pollution control or degradation, biodiversity conservation, and sustainable resource management. By integrating genomics, computational tools, and artificial intelligence, bioinformatics enables the analysis of complex biological datasets, such as metagenomes and environmental DNA (deoxyribonucleic acid), to uncover microbial diversity, pollutant degradation pathways, and ecological resilience. High‐throughput sequencing technologies and multiomics integration provide novel insights into microbial communities and their functional roles in bioremediation and ecosystem monitoring. Predictive modeling further enhances our ability to simulate microbial behavior in contaminated environments and assess the long‐term impacts of biotechnological interventions. Despite increased progress, challenges remain in managing large‐scale data, fostering interdisciplinary collaboration, and developing user‐friendly bioinformatics platforms. Future directions emphasize the application of machine learning, sustainable resource management, and collaborative frameworks to bridge bioinformatics and environmental sciences. Unlike traditional descriptive reviews, this work provides a critical evaluation of the functional gaps between genomic potential and in situ microbial activity. It offers a novel synthesis of how multiomics integration and predictive modeling can move beyond species cataloging toward a more robust, evidence‐based framework for environmental sustainability.

## 1. Introduction

Environmental challenges such as climate change, pollution, biodiversity loss, and ecosystem degradation have intensified the need for innovative solutions that go beyond conventional approaches. Environmental biotechnology has emerged as a critical discipline for addressing these issues [1]. By applying biological systems and organisms (microorganisms, plants, and enzymes) [2], it restores the environment [3], manages natural resources [4], and enables sustainable development [5]. Key applications include utilizing microbes for wastewater treatment [6] and employing organisms for the bioremediation of contaminated soil and water [7, 8]. This field moves beyond traditional “end‐of‐pipe” pollution treatment toward preventative, green technologies [4].

However, the complexity of microbial communities and environmental interactions requires advanced computational tools to decode and interpret vast biological datasets. This is where bioinformatics plays a transformative role, enabling researchers to integrate molecular insights with ecological applications [9]. Bioinformatics is an interdisciplinary scientific field that develops computational methods [10] and software tools to acquire, store, analyze, and interpret large, complex volumes of biological data, such as DNA (deoxyribonucleic acid), RNA (ribonucleic acid), and protein sequences [11, 12]. It combines biology, computer science, mathematics, and statistics to understand biological processes and systems [13].

Over the past decade, bioinformatics has become central to environmental biotechnology, driven largely by high‐throughput sequencing and multiomics technologies [14]. It is now indispensable for environmental applications [15], enabling big data analysis in ecology [16] and conservation [17]. By integrating genomics [18] and computational tools [19], bioinformatics supports the development of bioremediation strategies [20], the enhancement of sustainable agriculture (drought‐resistant crops), and the modeling of climate change impacts to inform global management [21]. Despite these advances, the field remains fragmented, with many studies focusing narrowly on isolated technologies rather than cohesive, integrative frameworks.

Several critical gaps hinder the full realization of bioinformatics in environmental biotechnology [22, 23]. First, there is often a disconnect between genomic potential and actual microbial activity under real‐world conditions, limiting the translation of laboratory findings into field applications [24]. Second, data integration across omics layers remains inconsistent due to interoperability challenges and the absence of standardized frameworks [25]. Third, predictive models frequently operate as “black boxes,” offering limited ecological interpretability and validation [26]. Addressing these gaps is essential for developing robust, evidence‐based strategies that can guide sustainable environmental interventions.

Moreover, existing literature (such as [16, 27, 28]; and [29]) has contributed valuable insights into specific aspects of bioinformatics, highlighting the role of metagenomics in uncovering microbial diversity, the utility of proteomics in identifying pollutant‐degrading enzymes, and the promise of computational modeling in bioremediation design; however, these studies remain discipline‐specific, lacking a comprehensive synthesis that integrates sequencing technologies, multiomics, and predictive ecology. This fragmentation underscores the need for a systematic evaluation of integrative bioinformatics approaches to identify pathways for future innovation, as several existing reviews detail individual tools or omics applications in isolation, without addressing the cross‐talk among genomic potential, real‐time sequencing advancements, and predictive modeling. Consequently, this review provides a unique integrative perspective by critically evaluating the transition from “potential function” to “in situ activity” and identifying specific methodological bottlenecks.

**Figure 1:**
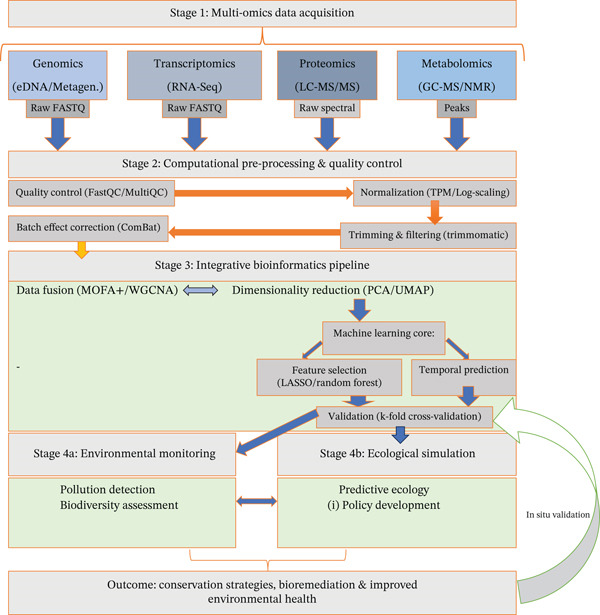
Schematic diagram illustrating the unified four‐stage integrative framework for environmental bioinformatics, tracing the workflow from initial ecosystem mapping to downstream environmental policy applications (constructed by the author).

While the unified four‐stage integrative framework (Figure 1) provides a robust conceptual blueprint, its practical implementation faces several technical bottlenecks. In Stage 1 and Stage 2, a primary challenge is data availability and heterogeneity, as environmental sampling often yields highly fragmented metagenomic data or incomplete metabolomic profiles due to low‐abundance analytes [30]. Furthermore, executing the integrative bioinformatics pipeline (Stage 3) introduces significant computational demands. Processing large‐scale multiomics matrices alongside machine learning (ML) cores requires high‐performance computing (HPC) architecture and specialized cloud infrastructure, which may limit accessibility in resource‐constrained research settings. Finally, the most critical bottleneck lies in field‐level validation (Stage 4a/4b) [31]. Bridging the gap between predictive ecological simulations (e.g., in silico metabolic modeling) and actual in situ bioremediation dynamics remains difficult, as fluctuating abiotic factors, such as seasonal pH swings, temperature shifts, and competing indigenous microbiomes, frequently disrupt laboratory‐derived predictions.

## 2. Integrative Bioinformatics Methodologies

### 2.1. Multiomics Integration Strategies

#### 2.1.1. Genomic and Metagenomic Analysis

Integrative bioinformatics approaches in environmental biotechnology encompass a wide array of techniques that are particularly impactful in genomic and metagenomic analyses, thereby enhancing our understanding of microbial communities and their roles in pollutant management [29, 32]. In microbial community studies, advanced sequencing technologies, such as high‐throughput DNA sequencing and metagenomic shotgun sequencing, allow researchers to explore the genetic diversity and functional potentials of microbial populations in various environments, from soil to aquatic ecosystems [33]. These studies enable the identification of specific microorganisms that thrive in contaminated sites, revealing how microbial communities respond to environmental stresses and interact with each other and their surroundings [34].

Furthermore, the insights gained from analyzing diversity indices and community composition can inform strategies for harnessing microbial capabilities in bioremediation [35]. When it comes to biodegradation and pollutant identification, metagenomic‐metabolomic analyses are particularly valuable, as they uncover the metabolic pathways and gene clusters involved in the degradation of specific pollutants, such as hydrocarbons. For instance, Li et al. [36] leveraged the Illumina HiSeq 6000 platform and SOAPdenovo for assembly to demonstrate how oil pollution upregulates specific monooxygenase and dioxygenase systems. By mapping unigenes to the KEGG database using Diamond software, the study uncovered a complete metabolic pathway degrading naphthalene into gentisic acid, whereas QIIME 2 and R (vegan/ggplot2) confirmed the recruitment of specialized genera like *Pseudomonas* and *Mycobacterium* to the contaminated site. By characterizing these microbial functions, researchers can target and optimize bioremediation efforts, ensuring that the selected microbial consortia are not only effective in degrading contaminants but also adaptable to fluctuating environmental conditions [37].

**Figure 2:**
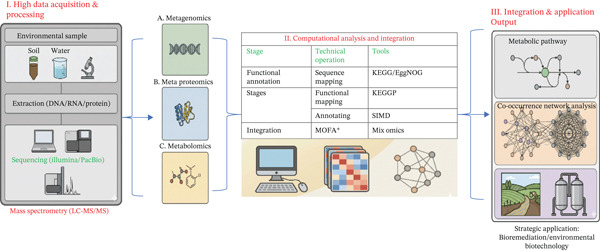
Modular pipeline for multiomics data integration (constructed by the author). Schematic representation of the transition from environmental sampling and multilayered data acquisition to computational functional mapping and strategic biotechnological applications.

Integrating multiomics, such as genomic data, with environmental monitoring tools enhances the ability to track microbial activity over time, assess the efficacy of bioremediation strategies, and develop predictive models for microbial responses to pollutants [38], as described in Figure 2. For instance, recent applications of H_2_O automated machine learning (AutoML) have successfully predicted optimal remediation variables, such as a 20–40‐day cultivation window for maximum efficiency, whereas genomic profiling has identified specific strains capable of near‐total (99.9%) removal of pollutants from soil matrices [39]. So, the application of integrative bioinformatics in environmental biotechnology not only deepens our understanding of microbial ecology but also paves the way for innovative solutions to address environmental challenges, ultimately contributing to ecological sustainability and pollution management [40].

Metagenomic approaches have transformed our ability to map microbial “dark matter,” yet a persistent gap remains between identifying genetic potential and confirming actual metabolic activity. Comparative studies [41, 42] show that DNA‐based snapshots often capture dead or inactive cells, leading to inflated estimates of functional capacity. Similarly, Ostos et al. [43] demonstrated that laboratory‐derived kinetic models fail to predict degradation rates in heterogeneous field environments, underscoring the challenge of translating controlled data into ecological reality. Collectively, these findings highlight a methodological bottleneck: the difficulty of linking gene clusters to measurable pollutant turnover in situ. To overcome this, future research must prioritize “active” metagenomics, integrating stable isotope probing (SIP) and metatranscriptomics, as Uhlik et al. [44] illustrated, to distinguish microbial presence from active metabolic contribution. This synthesis reveals that whereas sequencing technologies have expanded our knowledge base, functional validation remains the critical frontier for environmental bioinformatics, presenting interoperability hurdles fully detailed in Section 6.

### 2.2. Sequencing Technology

Sequencing technology has revolutionized the field of environmental biotechnology, driving advancements through high‐throughput sequencing methods that enable comprehensive analyses of biodiversity, genetic variation, and microbial responses to ecological stress [45]. To translate these massive datasets into effective bioremediation strategies, bioinformatics tools serve as the critical link between genetic sequences and functional application. Platforms such as MG‐RAST and QIIME 2 facilitate the high‐resolution taxonomic and functional profiling required to identify key microbial degraders and their underlying metabolic pathways. Furthermore, the integration of specialized databases like the EAWAG‐BBD allows for the precise prediction of xenobiotic degradation pathways, enabling the design of targeted and sustainable interventions for contaminated ecosystems [20, 46]. Moreover, high‐throughput sequencing technologies, such as Illumina sequencing and nanopore sequencing, provide unprecedented scalability and accuracy, allowing researchers to generate vast amounts of genomic data quickly and cost‐effectively [47]. This capability is instrumental in biodiversity monitoring [48], as it facilitates the assessment of species richness and abundance in various ecosystems, thereby informing conservation efforts and ecological assessments [49].

Additionally, these technologies allow for the detailed analysis of genetic variation within and between microbial populations, revealing how organisms adapt to changing environmental conditions and ecological stresses, such as climate change. Specifically, these tools enable the identification of functional gene clusters and strain‐level variations that drive community resilience, providing a high‐resolution map of microbial source tracking and ecosystem health that traditional cultivation methods cannot achieve [50]. By identifying specific genetic markers associated with resilience and stress response, researchers can better understand the mechanisms that underpin microbial survival and function in challenging habitats. Notably, these genomic signatures indicate that survival in extreme environments is often driven by metabolic rewiring and the rapid exchange of mobile genetic elements, providing a predictive framework for assessing an ecosystem′s self‐remediation potential under environmental pressure [51]. Ultimately, integrating cutting‐edge sequencing technologies into environmental biotechnology not only enhances our ability to monitor and manage biodiversity but also supports the development of innovative biotechnological applications to mitigate ecological challenges and promote ecosystem health.

The transition from short‐read platforms such as Illumina to long‐read technologies like Nanopore and PacBio has significantly improved genome assembly in complex microbial consortia, resolving many issues of fragmentation and incomplete assemblies. However, comparative analyses reveal persistent barriers: Long‐read sequencing still suffers from high error rates, and the cost of deep sequencing limits its routine use in environmental monitoring. Moreover, as Berg et al. [52] emphasized, the absence of standardized bioinformatics pipelines leads to variability in results across studies, undermining reproducibility and comparability. Collectively, these findings highlight that whereas scalability and resolution have advanced, methodological bottlenecks remain. Looking ahead, the future of environmental sequencing lies in portable, real‐time diagnostic kits, as demonstrated by reza Varzandi et al. [53], which reduce the “sample‐to‐result” lag time and enable adaptive management of contaminated sites. This synthesis underscores that technological innovation must be matched by cost reduction, error correction, and pipeline standardization to achieve reliable, field‐ready bioinformatics applications.

### 2.3. Data Integration and Management

Data integration and management are vital components of environmental biotechnology, particularly in the context of handling the vast and complex datasets generated from ecological and genomic studies [54, 55]. The establishment of databases tailored for environmental and genomic data enables researchers to systematically store, retrieve, and analyze information related to biodiversity, microbial functionality, and ecological conditions [56]. Research by Wang et al. [57] and Yoon et al. [58] demonstrates that structured data management is essential for addressing the “heterogeneity bottleneck,” where fragmented and nonstandardized data, ranging from unstructured experimental notes to complex genomic profiles, often impede actionable biological insight. A principal outcome of this study is that integrating disparate data types, such as eDNA signals and traditional field specimens, creates a synergistic effect that significantly improves the accuracy of identifying invasive species and monitoring pollutant degradation. Furthermore, the implementation of these integrated systems, supported by computational layers like generalized linear models (GLMs) to sieve environmental noise, has been shown to reduce analysis lead times from days to mere minutes while improving detection accuracy for contaminants to over 94%.

These databases serve as centralized repositories that not only facilitate data sharing among researchers but also enhance collaborative efforts in understanding environmental dynamics and evolutionary processes [59]. A key finding of their research is that these repositories allow partners to jointly analyze combined data across diverse areas and environmental gradients, which would otherwise remain fragmented. This collaborative framework is supported by expert‐based checks and automated consistency controls to ensure data integrity, which is essential for tracking complex biological responses to environmental changes. By integrating multisource data, researchers can better identify relevant datasets and “sieve environmental noise,” ultimately bridging the gap between raw data generation and actionable biological insights in biotechnology [59, 60].

Furthermore, the integration of multiomics data, encompassing genomics, transcriptomics, proteomics, and metabolomics, offers a robust framework for comprehensive environmental assessments [25]. By synthesizing data from these diverse omics layers, researchers can gain a holistic view of microbial interactions, metabolic pathways, and community responses to environmental stressors [25, 61]. Such integrative approaches allow for the identification of key functional genes and biomarkers that correlate with ecological health, thus enabling more accurate predictions of ecosystem responses to changes in biotic and abiotic factors [62]. Ultimately, effective data management and integration strategies are essential for advancing research in environmental biotechnology, facilitating the development of informed interventions and sustainable practices that promote ecological resilience and health.

Despite the proliferation of “omics” databases, the primary bottleneck in environmental biotechnology is no longer data generation but data interoperability. Comparative studies [30, 63] consistently show that repositories remain siloed, making it difficult to cross‐reference genomic data with geochemical and metadata parameters. This fragmentation limits the ability to derive holistic insights into microbial–environment interactions. Methodologically, the integration of multiomics layers is computationally demanding and often lacks a unified statistical framework to weigh the relative importance of different data types, such as transcript abundance versus protein expression [64]. Collectively, these findings highlight that whereas data volume has expanded dramatically, analytical coherence remains elusive. As elaborated in Section 6, addressing this analytical coherence requires the urgent development of standardized findable, accessible, interoperable, and reusable (FAIR) data schemas. Future efforts must therefore prioritize the development of standardized FAIR data schemas, as emphasized by Crystal‐Ornelas et al. [65], enabling seamless integration of biological datasets with real‐time environmental sensor networks. Such frameworks would transform fragmented data into actionable knowledge, bridging the gap between molecular discovery and ecological application.

### 2.4. Predictive Modeling

Predictive modeling is a crucial aspect of environmental biotechnology that leverages computational techniques to simulate and understand microbial behavior in contaminated sites and assess the impact of bioremediation strategies [66]. The integration of artificial intelligence (AI) and ML into bioremediation frameworks allows for the real‐time simulation of microbial growth kinetics and pollutant degradation paths. Their findings demonstrate that these advanced models can predict the efficacy of bioremediation strategies with high accuracy, specifically by identifying the most effective microbial consortia and optimizing the environmental conditions (such as pH and nutrient levels) required to accelerate the breakdown of persistent organic pollutants [67, 68].

By developing sophisticated models that incorporate ecological, genomic, and environmental data, researchers can predict how microbial communities will respond to various contaminants, optimizing the selection of microbial strains for bioremediation [69]. Their findings emphasize that multiomics data integration and advanced modeling can effectively map responses to contaminants and optimize microbial consortium assembly [70]. These models can simulate microbial interactions, metabolic activities, and population dynamics under varying conditions, providing insights into how different factors, such as nutrient availability and toxicant concentrations, may influence microbial effectiveness in degrading pollutants [71]. By integrating real‐time monitoring data with predictive models, researchers can refine their strategies and adaptations dynamically, ensuring a more targeted and effective approach to environmental remediation. Ultimately, predictive modeling not only enhances our understanding of microbial processes in contaminated environments but also aids in the design of innovative bioremediation technologies that can effectively restore ecological balance.

Predictive modeling offers a powerful blueprint for bioremediation, yet many current models are “black boxes” that lack biological interpretability or fail to account for the stochastic nature of natural ecosystems [72, 73]. Comparative studies highlight that while these models can forecast pollutant degradation under controlled conditions, their reliance on limited datasets and technological constraints often undermines ecological realism [71]. A significant methodological gap lies in the omission of evolutionary processes such as horizontal gene transfer (HGT) and viral–host interactions, both of which profoundly influence microbial adaptation and degradation efficiency [74]. Collectively, these limitations underscore the need for models that integrate mechanistic biology with probabilistic forecasting. Future directions point toward ML‐enhanced “digital twins” of contaminated sites, capable of assimilating multiomics data and environmental sensor inputs to deliver real‐time, adaptive predictions of remediation timelines under shifting climate scenarios. This synthesis reveals that while predictive modeling holds immense promise, its utility depends on bridging computational sophistication with ecological interpretability.

## 3. Specialized Databases, Software Tools, and Pipelines in Environmental Biotechnology

Integrative bioinformatics in environmental biotechnology relies on specialized databases that catalog the genetic and metabolic potential of microorganisms. For taxonomic identification and community profiling, researchers frequently turn to SILVA, RDP, and the Genome Taxonomy Database (GTDB), which provide high‐quality ribosomal RNA and genomic references [75]. When focusing on the breakdown of pollutants, databases like mibPOPdb and the University of Minnesota Biocatalysis/Biodegradation Database (UM‐BBD) are indispensable, as they map out the specific enzymatic reactions and catabolic pathways required to neutralize toxic compounds [76, 77]. Furthermore, platforms like MG‐RAST and MicrobeAtlas serve as massive repositories for metagenomic data, allowing scientists to compare microbial diversity across different global ecosystems and environmental conditions [78, 79]. Moreover, several tools and environmental applications are described in Table 1. In environmental biotechnology, bioinformatics pipelines serve as the bridge between raw biological data and actionable ecological insights. The process begins with data acquisition and quality control, where raw reads from platforms like Illumina or Oxford Nanopore are refined using tools such as FastQC [91], Trimmomatic [92], and Cutadapt [93]. This stage is vital for removing contaminants and low‐quality bases, ensuring that subsequent interpretations of microbial communities are not skewed by sequencing artifacts. Once cleaned, the data moves into assembly and binning, where tools like MEGAHIT [94] or metaSPAdes [95] reconstruct contigs into metagenome‐assembled genomes (MAGs). These MAGs are then rigorously validated for completeness and contamination using CheckM2 [96] or GUNC [97], followed by dereplication via dRep [98] to establish a nonredundant genomic catalog of the environment, as described in Figure 2.

**Table 1:** Key areas of application of integrative bioinformatics approaches in environmental biotechnology.

Area	Function and mechanism	Key tools/platforms	Advantages and limitations	Real‐world application and outcomes	References
Genomic and metagenomic analysis	Assesses microbial diversity and genetic potential via shotgun or targeted sequencing	QIIME 2, MG‐RAST, Mothur	Adv: Uncovers unculturable taxa. Lim: Cannot distinguish between live/dead cells or active/dormant genes	Identified 50+ novel hydrocarbon‐degrading gene clusters in oil‐contaminated marine sediments	Bolyen et al., 2019 [80]; Dombrowski et al., 2016 [81]
		Anvi′o	Adv: Links metabolic functions to specific taxa. Lim: Assembly struggles with high‐strain diversity	Identified specific bacterial strains for pollutant degradation in landfills	Eren et al., 2015 [82]
Sequencing technology	High‐throughput parallel processing of DNA/RNA for real‐time biodiversity monitoring	Illumina (HiSeq), Oxford Nanopore (MinION)	Adv: High‐speed data generation; long‐read capability (Nanopore). Lim: High error rates in long reads; high computational demand	Deployment of portable Nanopore devices for on‐site eDNA tracking of endangered aquatic species	Gygax et al., 2025 [83]; Winding et al., 2019 [84]
		PacBio HiFi	Adv: High‐accuracy long reads allow for complete circularized genomes. Lim: High capital cost and sensitive to DNA quality	Successfully assembled “dark matter” genomes from deep sea hydrothermal vents	Han et al., 2024 [85]
Data integration and management	Centralized organization of multiomics data for comparative analysis	NCBI, Earth Microbiome Project (EMP)	Adv: Facilitates global collaboration. Lim: Data heterogeneity; lack of standardized metadata formats	Integrated global soil datasets to map the distribution of antibiotic resistance genes (ARGs) across continents	Zhao et al., 2025 [86]; Zheng et al., 2022 [87]
		KBase	Adv: Adheres to FAIR data principles for global collaboration. Lim: Data storage bottlenecks for TB‐scale datasets	Created a global map of microbial functional diversity across ocean depths	Arkin et al., 2018 [88]
Predictive modeling	Employs unstructured models (e.g., Haldane and Monod) and mass/energy balances to simulate microbial growth and substrate degradation kinetics under toxic pressure	Mathematical kinetic models and specialized bioreactor systems	Adv: Proactive intervention; reduces lab costs. Lim: Highly dependent on the quality of training data (“garbage in, garbage out”)	Predicted phenol removal with high accuracy; 86%–99% removal efficiency in industrial	Priyadarshini et al., 2021 [89]
		Support vector machine (SVM), LSTM, RF	Adv: Simulates “what‐if” scenarios for bioremediation. Lim: Risk of overfitting where models fail in noncontrolled environments	Carbon emission prediction	Zhao et al., 2023 [90]

**Figure 3:**
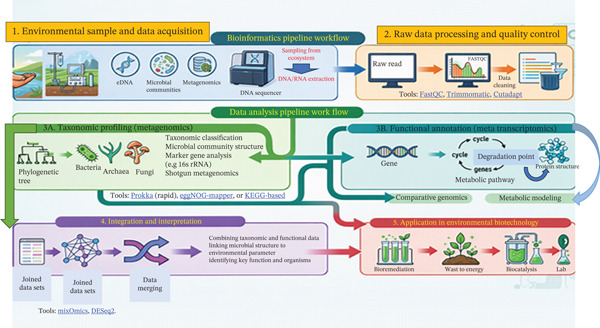
Workflow of bioinformatics pipelines in environmental biotechnology (constructed by the author). This workflow outlines the transition from (1) environmental sampling and multiomics acquisition to (2) raw data quality control, followed by (3) parallel taxonomic profiling and functional annotation. The process concludes with (4) multimodal data integration to drive (5) strategic applications in environmental biotechnology, such as bioremediation and biocatalysis.

The subsequent phase focuses on taxonomic profiling and functional annotation, utilizing GTDB‐Tk to assign precise lineages and platforms like Prokka [99] or eggNOG‐mapper [100] to map biological pathways. This step is crucial for linking microbial identity to specific ecological functions, such as nutrient cycling or bioremediation. A comprehensive list of these specialized pipeline tools, along with their respective advantages, limitations, and real‐world applications, is consolidated in Table 1. To achieve a holistic understanding, researchers employ multiomics integration and metabolic modeling, using tools like mixOmics [101] and DRAM [102] to combine genomic data with transcriptomics or proteomics. This systems‐level approach allows for the prediction of complex species interactions and metabolic capabilities. Finally, these workflows find critical applications in environmental systems, where they are used to monitor wastewater treatment efficiency, optimize anaerobic digestion (AD) for waste‐to‐energy, and track the degradation of pollutants in contaminated soil and water, as described in Figure 3.

## 4. Key Applications of Integrative Approaches in Environmental Biotechnology

### 4.1. Bioremediation: Identifying and Optimizing “Superdegraders”

Integrative bioinformatics transforms bioremediation from a trial‐and‐error process into a precision‐engineered strategy. By utilizing metagenomic data from specialized resources like the Plastic‐MBR database, researchers can identify specific bacterial taxa—for PET or *Bacillus cereus* for LDPE—that possess the unique genetic potential to degrade synthetic polymers [103, 104]. Computational tools like AlphaFold2 and MG‐RAST allow for the structural characterization of microbial enzymes to predict their catalytic efficiency against complex pollutants [20, 105]. Furthermore, in silico metabolic modeling and CRISPR‐Cas9 gene editing enable the design of “superdegraders” optimized for high‐resilience performance in heavy metal–contaminated soils [106, 107].

### 4.2. Waste‐to‐Energy: Optimizing AD and Bioelectrogenesis

In the waste‐to‐energy sector, bioinformatics provides a blueprint for managing the complex microbial consortia involved in AD and microbial fuel cells (MFCs) [108]. Integrating microbial electrolysis cells (MECs) into AD systems can increase methane production rates by up to 1.7 times through the enhancement of electroactive microbial selectivity [109]. Researchers use metatranscriptomics to monitor the real‐time expression of electrochemically active microbes (EAMs), which are critical for stable electricity generation in MFCs [110]. By mapping these metabolic fluxes, it is possible to optimize the utilization of volatile fatty acids from sludge, turning low‐concentration organic waste into clean bioelectricity and biogas.

### 4.3. Phytoremediation Enhancement: Decoding the Plant‐Microbe Interactome

Enhancing phytoremediation requires a deep understanding of the “cross‐talk” between hyperaccumulator plants and their associated rhizosphere microorganisms [111]. Integrative omics approaches characterize the proteins and metabolites in plant growth‐promoting rhizobacteria (PGPR) that alleviate metal phytotoxicity through mechanisms like chelation and redox reactions ([112]). Bioinformatics models analyze root exudate profiles to determine how specific plants recruit beneficial microbes to facilitate the uptake and stabilization of toxic elements [113]. This “smart phytoremediation” technology uses genomic insights to engineer plant‐microbe pairings that thrive in highly contaminated soils, significantly speeding up the detoxification of heavy metals and xenobiotics.

### 4.4. Biosensors: Engineering Intelligent “Whole‐Cell” Bioreporters

The development of whole‐cell biosensors (WCBs) relies on the bioinformatics‐driven identification of sensitive regulatory proteins that respond to specific environmental toxins. For instance, synthetic genetic circuits have been engineered to detect ionic arsenic at nanomolar concentrations, utilizing either fluorescent (Mer‐RFP) reporter proteins for real‐time monitoring [114, 115]. Advanced bioinformatics tools allow for the integration of Boolean logic gates into these cellular systems, enabling a single biosensor to process complex, multipollutant signals [116]. These portable, cost‐effective devices can be directly integrated onto nanoscaled chips for on‐site toxicity assessment in aquatic and terrestrial environments.

## 5. Case Study: Bioinformatics‐Aided Microbial Approach for Bioremediation of Wastewater Containing Textile Dyes

Azo dyes are recalcitrant and possess complex chemical structures that require multienzyme degradation pathways [117]. This case study was selected as a primary representative example because it perfectly operationalizes the concept of “integrative bioinformatics” to solve a classic problem in environmental biotechnology: the persistent challenge of multicomponent toxic waste. Moreover, this case was selected because it spans the entire workflow: from environmental sampling (wet lab) to integration of genomics/proteomics (GenPept) and structural bioinformatics (homology modeling/docking). Initially, microbial consortia were isolated from textile effluents and screened for their decolorization potential against reactive dyes such as Reactive Yellow F3R and Remazol Red RR. To move beyond descriptive observation, researchers employed an integrative genomic‐proteomic pipeline: Enzyme sequences (laccases and azoreductases) were retrieved from the GenPept Databank and subjected to homology modeling to resolve their 3D architectures. This critical step translated 1D sequence data into functional 3D models, allowing for molecular docking simulations to evaluate binding affinities and catalytic orientation between the dyes and microbial enzymes. These computational predictions were directly validated by spectroscopic evidence of dye cleavage, providing a mechanistic understanding of the degradation pathways [118].

Furthermore, the integration of metagenomic sequencing identified the specific functional taxa within the consortia responsible for breaking down the azo bonds [119]. When coupled with metatranscriptomic and metabolomic analyses, a clear activation of reductive pathways was observed, consistent with the predictive metabolic models discussed in Section 2.4 [117]. By linking these omics‐derived insights with simulations of enzyme kinetics, the study demonstrates how multilayered data integration can forecast degradation efficiency under fluctuating environmental conditions.

While challenges remain regarding the scalability of these models in nonsterile industrial settings and the limitations of template‐based protein modeling, this case serves as a robust proof of concept. It underscores how the alignment of predictive modeling, digital ecosystem twins, and multiomics validation can translate isolated laboratory findings into scalable, bioinformed remediation strategies [120].

## 6. Current Challenges

Integrative bioinformatics in environmental biotechnology faces critical hurdles. One of the most pressing issues is multiomics heterogeneity. The integration of genomic, transcriptomic, proteomic, and metabolomic datasets remains complex because each omics layer operates on different data scales, noise levels, and preprocessing standards. For instance, whereas genomic data are largely discrete and sequence‐based, metabolomic profiles are continuous and highly sensitive to environmental fluctuations. The absence of standardized normalization and batch correction protocols often leads to inconsistent results, making cross‐study comparisons unreliable. This heterogeneity limits the ability to construct unified models that accurately represent microbial functions in natural ecosystems [121]. A second major challenge involves ethical and regulatory constraints surrounding data sharing and genetic resource management. As environmental bioinformatics increasingly relies on global datasets, the governance of genetic information across borders has become a sensitive issue [122].

The third challenge is computational scalability. The exponential growth of environmental datasets—ranging from terabytes of metagenomic sequences to real‐time sensor data—demands HPC and cloud infrastructure [123]. Collectively, these challenges underscore the need for methodological innovation and global collaboration. Addressing data heterogeneity through FAIR standards, harmonizing ethical frameworks for genetic data governance, and expanding equitable access to computational resources will be essential for advancing integrative bioinformatics toward truly sustainable environmental management.

## 7. Future Directions (2025 and Beyond)

Future directions in environmental biotechnology from 2025 and beyond are set to be revolutionary, transitioning from descriptive cataloging toward prescriptive engineering, driven by advances in various multimodal technologies. To maintain scientific rigor, these emerging tools must be distinguished by their current operational maturity, separating active computational workflows from speculative paradigms.

Empirical and active implementations: Several advanced architectures have moved beyond purely theoretical design and are actively deployed in exploratory research or pilot environments. Researchers are increasingly utilizing generative AI models, such as ProteinMPNN and AlphaFold3, to engineer new carbon‐capture materials and synthetic microbial pathways aimed at degrading recalcitrant toxins that are not naturally occurring. Simultaneously, a shift toward single‐cell genomics addresses the limitations of bulk metagenomics by enabling the investigation of individual microbial interactions within complex biofilms, fostering more targeted treatment strategies. Additionally, current pilot systems are beginning to employ Edge AI and cloud‐based platforms for real‐time analysis of environmental sensors, delivering immediate feedback for pollution control efforts and reducing the latency of traditional laboratory‐based monitoring.

Conceptual and theoretical frontiers: Conversely, other highly anticipated paradigms remain largely conceptual due to fundamental constraints in hardware scaling and environmental unpredictability. For instance, the deployment of AI‐powered “digital twins” of entire macroecosystems remains a theoretical goal. While remote sensing and real‐time IoT networks provide localized data streams, fully synthesizing these into a responsive ecosystem‐scale digital twin is currently bottlenecked by the unpredictable chaotic variables of shifting climates and multitrophic biological networks. Similarly, the integration of quantum machine learning (QML) to solve “intractable” protein folding problems and simulate multispecies molecular docking kinetics is fundamentally restricted by hardware limitations. While theoretically poised to outperform classical molecular dynamics (MD) simulations by utilizing quantum parallelism to navigate vast chemical spaces, practical QML applications remain constrained within the experimental limits of noisy intermediate‐scale quantum (NISQ) processors. Consequently, these quantum and macroscale modeling approaches serve as foundational roadmaps for future decades rather than deployable computational frameworks for immediate environmental engineering.

## 8. Ethical Considerations and Regulatory Frameworks in Environmental Bioinformatics

While integrative bioinformatics offers transformative potential for environmental restoration, its application is inextricably linked to complex ethical and regulatory landscapes that demand careful navigation. Ethically, the field must balance the “precautionary principle” against the urgency of environmental crises, ensuring that engineered microbial interventions do not lead to unintended ecological disruptions or the loss of native biodiversity [20]. Furthermore, the digitalization of genetic resources has sparked critical debates surrounding digital sequence information (DSI) and indigenous data sovereignty, highlighting the need for equitable benefit‐sharing and protection against digital biopiracy. From a regulatory perspective, the lack of global harmonization, evidenced by the disparate “product‐based” versus “process‐based” oversight models, creates significant hurdles for the cross‐border deployment of biotechnological solutions. Ultimately, for bioinformatics to achieve its full potential in environmental biotechnology, it must operate within a robust framework that prioritizes biosecurity, environmental justice, and transparent governance.

## 9. Conclusion

This review has articulated a unified integrative framework for environmental bioinformatics, conceptualizing it not as a collection of isolated sequencing or modeling tools but as an unbroken, iterative pipeline transitioning from potential function to in situ activity. Bioinformatics has emerged as a cornerstone of environmental biotechnology, enabling the systematic exploration of microbial communities, pollutant degradation mechanisms, and ecological dynamics through advanced computational and sequencing technologies. Its integrative approaches—spanning genomics, data management, and predictive modeling—have redefined strategies for bioremediation, biodiversity monitoring, and sustainable agriculture. However, the field must overcome persistent challenges, including the complexity of big data, the need for interdisciplinary expertise, and the demand for accessible analytical tools. Looking ahead, AI and ML will play pivotal roles in predictive analytics and resource optimization, whereas collaborative networks between bioinformatics and environmental science will strengthen translational outcomes. Continued innovation and investment in integrative bioinformatics are essential to harness its full potential, ensuring that environmental biotechnology contributes effectively to global sustainability and ecological resilience. Ultimately, this review establishes that environmental biotechnology must transition from descriptive cataloging to predictive engineering through a unified four‐stage framework. By linking multiomics data acquisition (Stage 1) and quality processing (Stage 2) with an integrative ML core (Stage 3), researchers can reliably drive real‐time environmental monitoring and ecological simulations (Stages 4a/4b), as conceptualized in Figure 1.

## Author Contributions

Conceptualization, writing—original draft, and visualization were performed by Yohannes Tsegay Teklay.

## Funding

No funding was received for this manuscript.

## Disclosure

The author has read and approved the final manuscript.

## Ethics Statement

The author has nothing to report.

## Consent

The author has nothing to report.

## Conflicts of Interest

The author declares no conflicts of interest.

## Data Availability

All data generated or analyzed during this study are included in this published article.
